# TOLLIP Protein Expression Predicts Unfavorable Outcome in Renal Cell Carcinoma

**DOI:** 10.3390/ijms232314702

**Published:** 2022-11-25

**Authors:** Adam Kowalewski, Damian Jaworski, Jędrzej Borowczak, Mateusz Maniewski, Krzysztof Szczerbowski, Paulina Antosik, Justyna Durślewicz, Marta Smolińska, Joanna Ligmanowska, Dariusz Grzanka, Łukasz Szylberg

**Affiliations:** 1 Department of Tumor Pathology and Pathomorphology, Oncology Centre Prof. Franciszek Łukaszczyk Memorial Hospital, 85-796 Bydgoszcz, Poland; 2 Department of Clinical Pathomorphology, Collegium Medicum in Bydgoszcz, Nicolaus Copernicus University in Torun, 85-094 Bydgoszcz, Poland; 3 Division of Ophthalmology and Optometry, Department of Ophthalmology, Collegium Medicum in Bydgoszcz, Nicolaus Copernicus University in Torun, 85-067 Bydgoszcz, Poland; 4 Department of Obstetrics, Gynaecology and Oncology, Chair of Pathomorphology and Clinical Placentology, Collegium Medicum in Bydgoszcz, Nicolaus Copernicus University in Torun, 85-094 Bydgoszcz, Poland

**Keywords:** TOLLIP, renal cell carcinoma, kidney, cancer, expression, prognosis, survival

## Abstract

Resistance to systemic therapy is one of the hallmarks of renal cell carcinoma (RCC). Recently, TOLLIP has emerged as a possible driver of autophagy and chemoresistance. We explored the relationship between primary and metastatic RCC tumor characteristics, patient survival, and TOLLIP expression. The tissue microarrays cohort contained 95 cores of the primary tumor, matched metastases, and matched adjacent tissues derived from 32 RCC patients. TOLLIP expression in tumor samples was evaluated using the H-score. All examined samples showed cytoplasmic TOLLIP expression, with a median value of 100 in primary tumors, 107.5 in metastases, and 220 in the control group. The expression was significantly higher in the normal adjacent tissues compared to primary or metastatic RCC (*p* < 0.05). We found a positive correlation between expressions of TOLLIP in the primary tumor and its metastases (*p* < 0.05; k = 0.48). TOLLIP expression significantly correlates with a lower overall survival rate (*p* = 0.047). TOLLIP functions as a ubiquitin-LC3 adaptor in the intracellular pathway associated with autophagy. Relative TOLLIP overexpression may augment autophagy-related signaling, limiting susceptibility to therapy. The blockade of TOLLIP physiological function seems to be a promising approach to overcoming resistance to systemic therapy.

## 1. Introduction

Renal cell carcinoma (RCC) is the tenth most common malignancy in women and sixth in men. It is responsible for respectively 5% and 3% of all oncological diagnoses worldwide and impacts over 400,000 individuals per year [1,2]. The survival rate ranges from >93% in stage I to 12% in the IV stage metastatic disease [3]. While localized tumors are successfully treated with partial or radical nephrectomy, the higher stages require systemic treatment with targeted immunotherapy or chemotherapy. Unfortunately, renal cancer (RCC) is known to develop resistance to chemotherapy, which results in a worse prognosis. Due to that fact, there is a strong emphasis on the study of new predicting markers and target points for the therapy of RCC [4]. Many different mutations are responsible for carcinogenesis in RCC, but 3p loss and Von Hippel Lindau (VHL) seem crucial in RCC. Clear cell RCC arises from proximal convoluted tubule cells and accounts for approximately 75% of cases [2,3,5]. Although RCC is an immunogenic tumor, there is mounting evidence that immune cells and inflammatory pathways enhance tumor growth and immune escape. However, recent studies are beginning to uncover the mechanisms of immune escape in RCC and explain the role of the inflammatory process.

Recently, toll-interacting protein (TOLLIP) has emerged as a potential therapeutic target and prognostic marker due to its links with the pathways regulating autophagy and participating in the development of chemoresistance. TOLLIP is encoded by a TOLLIP gene localized on the 11th chromosome [6]. It is composed of three distinct domains—the target of the Myb (TOM) binding domain (TBD), the conserved 2 (C2), and the coupling of ubiquitin to ER degradation (CUE). The TBD is responsible for the recruitment and binding of TOM1 and endosomal sorting. The C2 is involved in phosphoinositide binding and placing protein on membranes and contains LC3-interacting regions (LIRs), which are crucial for autophagy. The CUE domain binds the surface of TOLLIP to proper receptors [7,8]. The protein is implicated in signaling modulation of TLR 2 and 4, transcription growth factor (TGF)-β, and interleukin 1 receptor (IL-1R1), and TOLLIP participates in pathways connected to interleukin (IL)-1β and IL-13 [8,9,10,11,12,13,14].

In this study, we evaluate TOLLIP expression, investigate its correlations with clinicopathological features of renal cell carcinoma, and explore its potential use as a prognostic marker and the target point for targeted immunotherapy in RCC.

## 2. Results

### 2.1. IHC Analysis

The study included 95 tissue microarray cores derived from 32 RCC patients. During the IHC staining procedure, 3 cores were lost. The characteristics of the TMA cohort are presented in Table 1. The median follow-up was 105 months.

**Figure 1:**
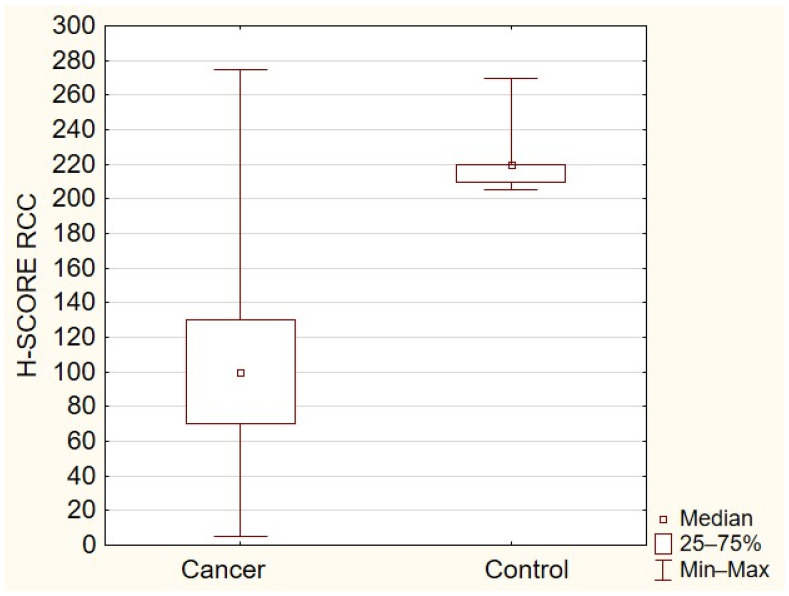
TOLLIP expression in RCC and control (*p* < 0.05).

**Figure 2:**
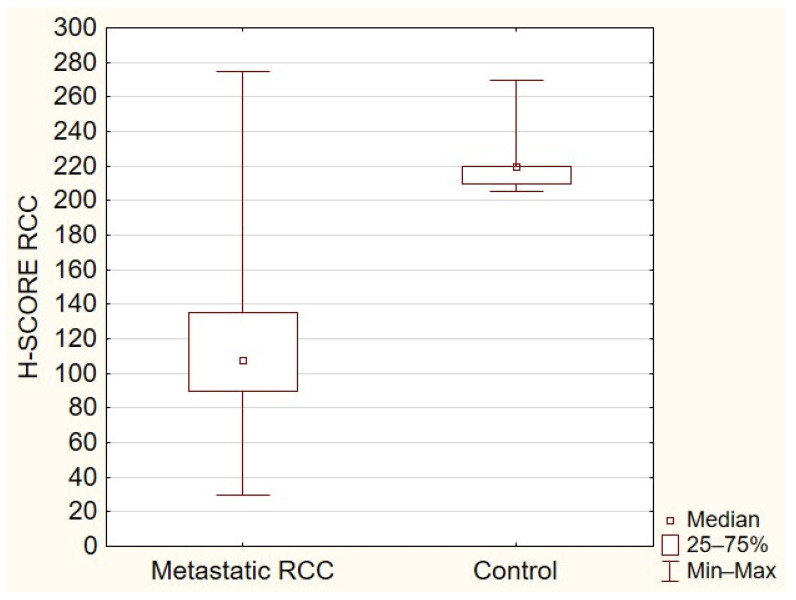
TOLLIP expression in metastatic RCC and control (*p* < 0.05).

**Figure 3:**
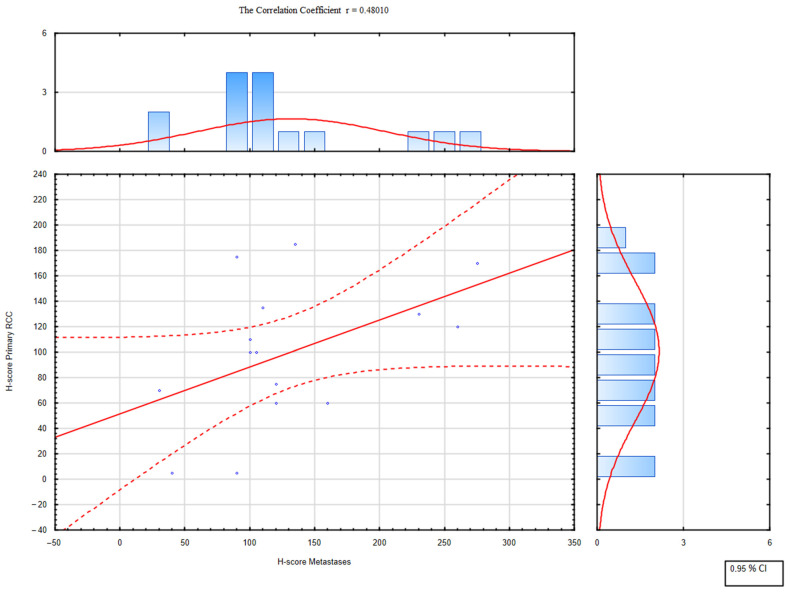
Correlation between TOLLIP expression in primary and metastatic RCC.

All the tissue samples presented cytoplasmic TOLLIP expression with the median expression of TOLLIP in the primary tumor at 100 (interquartile range 5–210), in metastatic tissue at 107.5 (interquartile range 30–275), and at 220 in the control group (interquartile range 205–270). The expression of TOLLIP was significantly higher in normal adjacent tissues compared to primary (Figure 1) or metastatic RCC (*p* < 0.05) (Figure 2). The analysis demonstrated a moderate positive correlation between expressions of TOLLIP in primary tumors and their metastases (*p* < 0.05), with a correlation coefficient of 0.480 (Figure 3).

**Figure 4:**
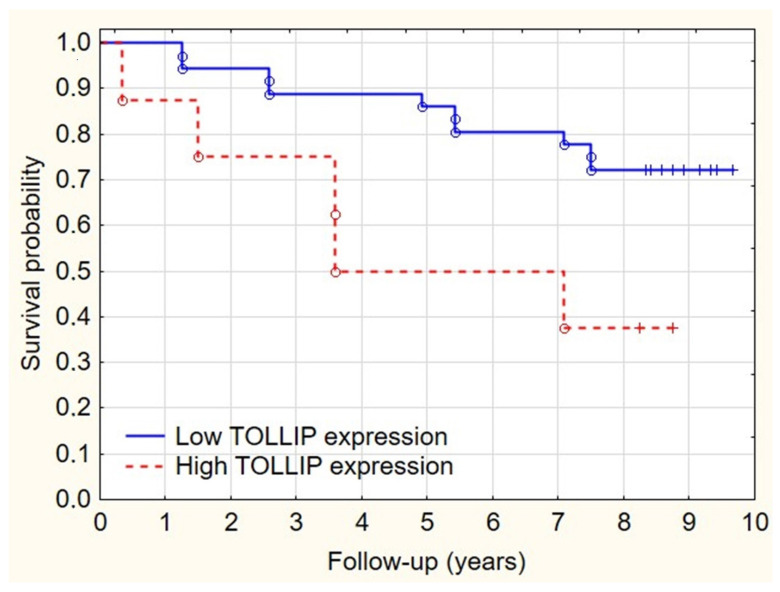
Survival differences in high and low TOLLIP expression groups.

**Figure 5:**
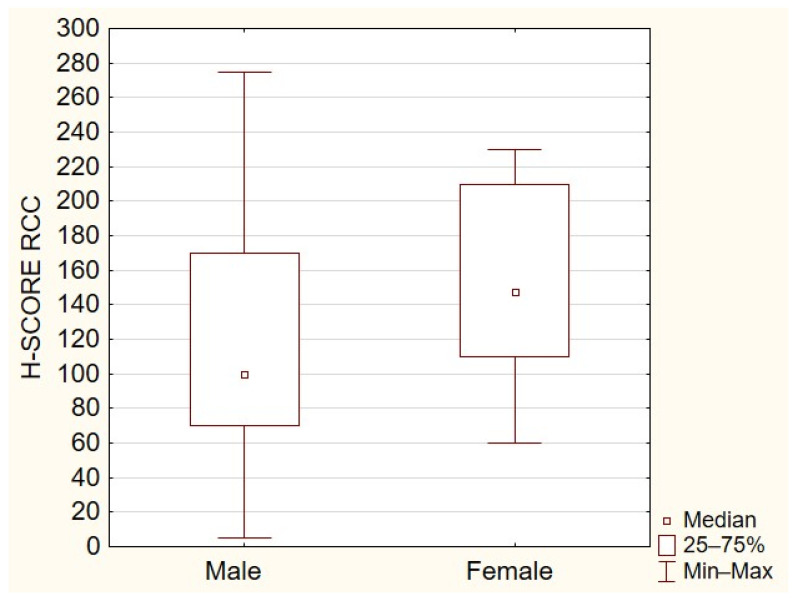
The difference in TOLLIP expression depends on sex (*p* < 0.05).

The overexpression of TOLLIP significantly correlated with worse overall survival (*p* = 0.047) (Figure 4). In the analyzed group, tissues collected from female patients revealed significantly higher TOLLIP expression (*p* < 0.05) (Figure 5).

We found no significant correlation between TOLLIP expression, lymph nodes infiltration, and the presence of renal capsule or vein invasion.

### 2.2. In Silico Analysis

**Figure 6:**
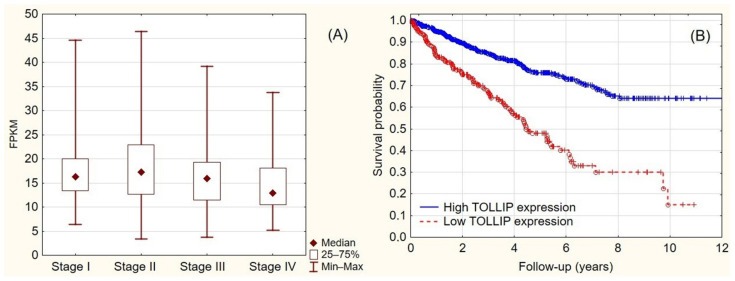
(**A**) TOLLIP expression in the TCGA RCC cohort depends on stage; (**B**) survival differences in the TCGA RCC groups depend on TOLLIP expression.

TOLLIP expression was analyzed in 877 RCC cases acquired from the TCGA database (Table 2) [15]. The samples were assigned to low-TOLLIP or high-TOLLIP groups based on the established cutoff (12.48 FPKM). Of the total, 216 (24.63%) RCCs showed low TOLLIP expression, while in 661 (75.37%) RCCs the expression of TOLLIP was high. High TOLLIP expression was observed in 367 (81.37%) stage I tumors, 79 (77.45%) stage II tumors, 129 (68.98%) stage III tumors, and 55 (53.4%) stage IV tumors. TOLLIP expression was the lowest in stage IV tumors (*p* < 0.005) (Figure 6A).

Kaplan–Meier survival analysis of the TCGA data revealed that TOLLIP expression positively correlated with patients’ overall survival (median OS for low vs. high expression = 950 vs. 1136 days; *p* < 0.0001). Low TOLLIP expression was predictive of shorter patient survival (HR = 2.85 [2.19–3.82]; *p* < 0.0001) (Figure 6B).

**Figure 7:**
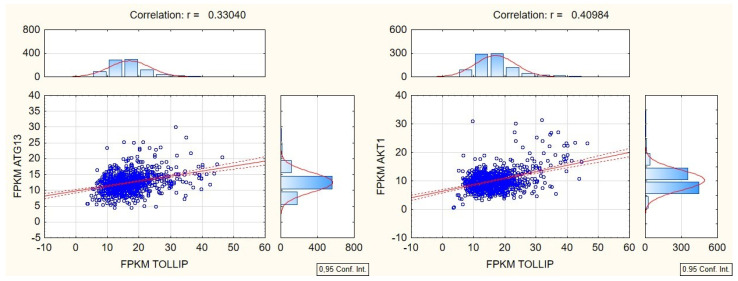
The correlation between the expression of TOLLIP and ATG13 (**left**) and between TOLLIP and AKT1 (**right**) in the TCGA cohort.

The TCGA cohort was then investigated for potential correlations between the expression of TOLLIP and proteins that partake in autophagy. The results are summarized in Table 3. The most notable was the positive correlation with ATG13 (autophagy-related protein 13), MAP1LC3 (LC3 coding gene), TAX1BP1 (TAX1-binding protein 1), and AKT1 (Figure 7). TOLLIP expression is also negatively correlated with STAT3 and VMP1 expression (*p* < 0.05) (Figure 7).

## 3. Discussion

### 3.1. TOLLIP in Cancers

TOLLIP functions as a negative regulator of inflammatory signaling, cell turnover, and immune surveillance [33]. Recently, its role in carcinogenesis has been extensively studied; however, different patterns of expression in cancers suggest that local factors may influence its prognostic value. In breast cancer, the expression of TOLLIP was heterogeneous; in 25% of tumors, the expression was higher than in normal tissue, but in 35%, the expression was lower than in the control and increased during breast cancer progression. It is likely attributed to the accumulation of other molecular determinants, including the mutation of p53 and polyclonal cancer expansion [34]. In triple-negative breast cancer, TOLLIP-mediated autophagy can hinder disease progression via the degradation of transmembrane protein 63A (TMEM63A), a novel oncogene that promotes cancer cell proliferation in triple-negative breast cancer [35].

Dysregulations of toll-like receptor (TLR) signaling have been associated with multiple inflammatory-related cancers. Variable TOLLIP expression was found in colitis-associated cancer (CAC). The development of CAC is associated with chronic inflammation, driven by the overstimulation of the IL-6/NF-κB pathway and the activation of the JAK/STAT3 pathway. In response to IL-1R activation, TOLLIP disrupts NF-κB signaling, impairing CD4+ and regulatory T cell infiltration and protecting mice against acute colitis [36]. TOLLIP also inhibits STAT3 signaling, but the mechanism of its action is still unclear [37]. STAT3 is known to mediate autophagy through the Bcl2-Beclin1 complex. In cervical cancer, STAT3 was overexpressed and negatively correlated with LC3B level. Its knockdown augmented autophagy and decreased the proliferation and migration of cervical cancer cells in vitro and in vivo [38].

In hepatocellular carcinoma (HCC), TOLLIP expression was upregulated, associated with more rapid tumor growth in vivo, enhanced HCC cell migration, and proliferation in vitro. It also promoted the epithelial–mesenchymal transition (EMT), invasiveness, and metastasis, likely through the activation of PI3K/AKT/mTOR signaling [39].

In colorectal cancer models, TOLLIP deficiency resulted in neutrophil reprogramming, increasing tumor immune surveillance, T cell activation, and reducing tumor burden. The knockdown of TOLLIP increased STAT5 and reduced STAT1 signaling, which are responsible for the expression of CD80 and PD-L1, respectively. Higher levels of TGF-β and lower levels of IL-1β, CD14, and CCR5, markers of circulating neutrophils, suggest that TOLLIP deficiency may facilitate the resolution of chronic inflammation during colon carcinogenesis [36]. In contrast, TOLLIP ablation in mice resulted in reduced tumor incidence, smaller lesions, and reduced tumor cell turnover, suggesting that high TOLLIP levels favor the development of CAC [34]. TOLLIP overexpression antagonizes TGF-β-mediated transcription and EMT by accelerating the ubiquitination and degradation of activated TβRI. For the negative regulation of the TGF-β signaling pathway, cooperation between Smad7 and TOLLIP is required [13].

### 3.2. TOLLIP, Autophagy, and Cancers

Autophagy plays an important role in each stage of cancer progression. Depending on environmental stimuli, it can modulate EMT, facilitate the metabolic switch to aerobic glycolysis, limit immunological response, influence cell survival, and promote cancer progression [40,41,42]. TOLLIP functions as a ubiquitin-LC3 adaptor in the intracellular pathway associated with autophagy [43]. It binds to ubiquitinated protein aggregate and bridges the complex with LC3, a central protein in autophagy and autophagosome biogenesis [44]. During the aggrephagy, the selective degradation of protein aggregates, TOLLIP is responsible for the recruitment of autophagy receptors [43]. In TOLLIP-deficient cells, the formation of autophagosomes is impaired [45]. TOLLIP overexpression seems to clear human cells of cytotoxic proteins containing glutamine repeats (polyQ) associated with Huntington’s disease by autophagy, while in TOLLIP-deficient cells the aggregation of polyQs was reported [43]. Upon mitochondrial stress, TOLLIP is required to ensure the trafficking to the lysosome [46]. TOLLIP knockdown in macrophages disrupts endosomal–lysosomal fusion, vesicular trafficking, and autophagosome formation, implying that TOLLIP signaling is crucial for the functioning of multiple autophagy pathways [47].

TOLLIP’s involvement in protein trafficking and degradation may be even more important when intercellular and intracellular signaling pathways are dysregulated [13]. However, the mechanism of TOLLIP interactions remains unclear. In the setting of high LPS stimuli, TOLLIP limits the inflammatory response, relieves the burden of the acute immune response, inhibits NF-κB and IL-1 signaling, and prevents excessive tissue damage [48]. When the amount of LPS is low, it facilitates the maintenance of chronic inflammation, which may favor carcinogenesis [49].

### 3.3. TOLLIP in Renal Cancer

The role of TOLLIP in RCC is still unclear. In this study, TOLLIP expression was downregulated compared to adjacent normal tissue, and its high expression correlated with worse overall survival. However, the analysis of the TCGA cohort revealed an opposite trend. Those disparities can arise due to the high heterogeneity of RCC and the generalizability of the TCGA cohort [50]. TOLLIP can exist in the form of numerous alternate transcripts, which generate protein variants that may have altered N-terminal domains, altered TBD-loop-coil domains, or a truncated C2 domain. As a result, local TOLLIP polymorphism may affect the intensity of immunohistochemical staining [51].

In the TCGA cohort, the percentage of tumors with high TOLLIP expression decreased in line with an increasing stage (*p* < 0.05). Those results are compatible with reports, suggesting that, in the early stages of carcinogenesis autophagy, which TOLLIP is an adaptor of, tumor suppression may be exerted through the degradation of potentially tumorigenic proteins [52]. Wu et al. also reported that in the EC1 subtype of renal cell carcinoma, which is characterized by a worse prognosis, TOLLIP signaling is silenced and probably associated with the immune suppression phenomenon [53].

The analysis of TOLLIP correlations with other autophagy proteins sustains the predictive uncertainty (Table 3). In the TCGA cohort TOLLIP correlated with higher ATG13, higher TAX1BP1, and lower STAT3 levels, but also with higher ATK1 and lower VMP1 expression [54,55]. Those results indicate that TOLLIP may contribute to ATG13-mediated autophagosome formation [43,56]. Although ATG-13 is essential for autophagy initiation, its overexpression inhibits autophagy. Therefore, the results of its interaction with TOLLIP are hardly predictable and need further investigation [57]. TAX1BP1 and TOLLIP, due to their structural similarities, are referred to as SQSTM1-like receptors, and both can mediate the induction of autophagy [58]. A positive correlation between those proteins suggests that their activity is additive. STAT3 signaling inhibits autophagy on molecular and cellular levels. Since TOLLIP overexpression is associated with lower STAT3 levels, it may indicate enhanced autophagy [59].

In contrast, high TOLLIP expression correlates with high expression of Akt1 and VMP1, which inhibit autophagy by downregulating UVRAG or reducing vesicle trafficking and autophagosome formation, respectively [54,55]. In hepatocellular carcinoma, TOLLIP overexpression increased proliferation, invasion, and epithelial–mesenchymal transition, accelerating tumorigenesis via the activation of the PI3K/AKT pathway [39]. The effects were attenuated by TOLLIP blockade in HCC cells which was attenuated by TOLLIP silencing. Similarly, Akt1 inhibitors hindered the proliferation of hepatocellular carcinoma cell lines and induced autophagy-associated cell death [60]. Hence, the blockade of the TOLLIP–PI3K/AKT axis seems promising for the management of HCC. VMP1 is an endoplasmic reticulum resident protein that regulates interorganellar connectivity and autophagosome formation [61]. In glioma, high VMP1 levels predict poor patient prognosis and are associated with resistance to therapy [62]. The negative correlation between TOLLIP and VMP1 expression suggests that TOLLIP may suppress VMP1-related pathways (Table 3). Given the ambiguous result, the in vivo effect of increased TOLLIP expression may be determined by the local balance of other signaling pathways.

In later stages of carcinogenesis, autophagy enables tumor survival in a stressful environment [63]. That seems to be particularly important in renal cancer, due to the frequent overproduction of proangiogenic and proinflammatory cytokines, which disrupts the angiogenic balance through the stimulation of the RAS/RAF/MEK/ERK and PI3 Kinase/AKT/mTOR signaling pathways [64]. In later stages of the disease, TOLLIP knockdown may lead to increased IL-6/NF-κB activity and the activation of the protumorigenic JAK/STAT3 signaling [37]. Therefore, the underexpression of TOLLIP in the TMA group might have occurred during tumor immunoediting in the early stages of RCC when TOLLIP-mediated autophagy is still an efficient way of preserving the genome stability and preventing malignant transformation [63]. It seems feasible that the relative overexpression of TOLLIP is a manifestation of its residual activity, persisting after incomplete silencing. Such activity could be associated with increased autophagy in cancer cells, increased tolerance to unfavorable environments, and the occurrence of resistance to therapy [63,65,66].

### 3.4. Perspectives of TOLLIP-Targeted Therapy in RCC

**Figure 8:**
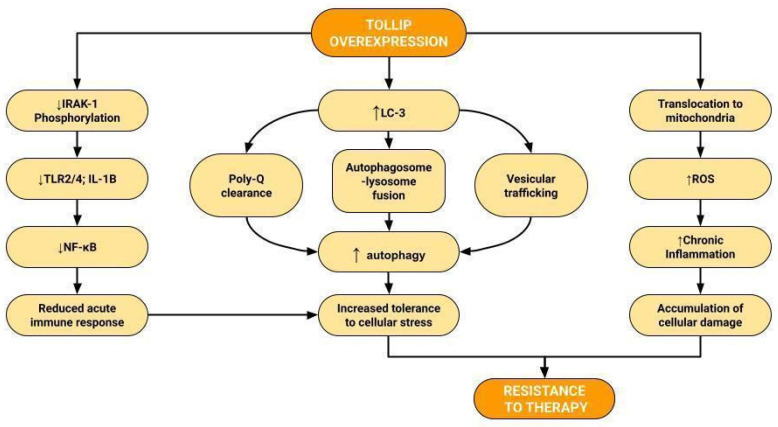
Effects of TOLLIP in renal cell carcinoma [8,43,49]. Overexpression of TOLLIP decreases IRAK-1 phosphorylation, reducing the activation of TLR2/4 and the secretion of IL-1B. It leads to decreased activity of NF-kB signaling and, in effect, reduced acute immune response. TOLLIP binds to LC-3, which stimulates autophagy by increased Poly-Q clearance, autophagosome–lysosome fusion, and vesicular trafficking. Augmented TOLLIP translocation to mitochondria increases ROS levels and sustains chronic inflammation. Reduced acute immune response and increased autophagy favor tolerance to cellular stress and the accumulation of cellular damage, which may cause resistance to therapy.

The role of autophagy in renal cancer is not yet fully explained. While some in vivo studies demonstrated that the induction of autophagy is capable of activating apoptosis in precancerous cells, other authors argue that the intensification of autophagy during chemotherapy may facilitate tumor survival and hence accelerate tumor progression [67,68,69,70]. Despite its dual role in carcinogenesis, autophagy has been recently suggested as a therapeutic target for renal cancer [4,69]. The rationale is based on the fact that RCC cells exhibit an elevated basal level of autophagy, can alleviate periods of therapy-induced stress, and promote drug and radiation resistance [66,71] (Figure 8). Currently, only pembrolizumab can be considered as adjuvant therapy for intermediate- or high-risk operable RCC [I, C], while the metastatic disease is managed with tyrosine-kinase inhibitors (TKIs) or TKIs in combination with a PD-L1/CTLA-4 blockade [72]. Hence, targeting autophagy by co-inhibiting TOLLIP signaling may be an important step in increasing the efficiency of other therapeutic agents. Autophagy inhibitors were shown to enhance the efficacy of TKIs, blocking VEGF signaling and impeding neoangiogenesis caused by the inactivation of the VHL gene [73,74]. The reduction of AKT/mTOR signaling was also augmented by autophagy blockade and could potentialize the effects of VEGF inhibitors and reduce the metastatic burden of RCC [75,76,77].

Currently, resistance to therapy is considered the leading problem in achieving effective systemic treatment in RCC. Although TKIs are a first-line treatment, eventually, most cancers become resistant [78]. Considering that autophagy inhibitors enhance the properties of currently used second-line agents, such as PD-L1 and mTOR inhibitors, TOLLIP seems to emerge not only as a prognostic factor but also as a potential therapeutic target in renal cell cancer.

## 4. Materials and Methods

### 4.1. Immunohistochemistry Staining

Immunohistochemical (IHC) staining was performed on a tissue microarray (TMA) slide preparation obtained from a commercial manufacturer (US Biomax, Rockville, MD, USA; TMA catalog number KD951a). The TMA slide contained specimens from 32 patients diagnosed with renal cell carcinoma (RCC) and matched normal adjacent tissue. Automated immunostaining for anti-TOLLIP antibody (HPA038621, Sigma–Aldrich, Merck KGaA, Darmstadt, Germany) was carried out with ultraView Universal DAB Detection Kit (Roche Diagnostics/Ventana, Tucson, AZ, USA) using BenchMark^®^ ULTRA (Roche Diagnostics/Ventana Medical Systems, Tucson, AZ, USA). The slide was deparaffinized and rehydrated in the EZ Prep solution, and then antigen retrieval was achieved in a high-pH cell-conditioning (CC1) solution. Next, the slide was incubated with primary rabbit polyclonal anti-TOLLIP antibody (1:400) for 32 min at RT. Subsequently, the tissue sections were counterstained with hematoxylin for 12 min and bluing reagent for 4 min. Tissue sections were dehydrated in increasing ethanol concentrations (80, 90, 96, and 99.8%) Finally, xylene was used to clear the sections, which were then cover-slipped in a medium (Dako, Agilent Technologies, Santa Clara, CA, USA).

### 4.2. Evaluation of Immunohistochemistry Staining

The stained tissue microarray was evaluated using the light microscope ECLIPSE E800 (Nikon Instruments Europe, Amsterdam, The Netherlands) at 20× original objective magnification by two independent pathologists. Images were scanned using the VENTANA DP 200 slide scanner (Roche Diagnostics/Ventana Medical Systems, Tucson, AZ, USA). The scoring system for cytoplasmic TOLLIP expression was determined by multiplying the staining intensity of cells and the percentage of cells at each staining intensity level. Staining intensity was graded as negative (0), weak (1+), moderate (2+), and strong (3+); thus, the H-score ranged from 0 to 300. Using the Cutoff Finder tool [79], we set the best cutoff at 135, and all the samples were classified as low (H-score < 135) or high (≥135) TOLLIP expression.

### 4.3. In Silico Analysis

Gene expression data for the cohort of 877 RCC patients were obtained from www.cBioPortal.org and UCSC Xena Browser (http://xena.ucsc.edu/, accessed on 9 September 2022). The RNA-sequencing (RNA-seq) datasets were normalized with the DESeq2 method. Data were categorized into low- and high-expression groups following the cutoff points determined in Evaluate Cutpoints software. The cutoff values for low and high TOLLIP mRNA expression were ≥12.58 and <12.58, respectively.

### 4.4. Statistical Analysis

Statistical analysis was performed using Statistica version 13.3 (StatSoft, Tulsa, OK, USA) and SPSS version 26.0 software (IBM Corporation, Armonk, NY, USA). A *p*-value of <0.05 was considered statistically significant.

## Figures and Tables

**Table 1:** Baseline characteristics of TMA (n = 32) patient cohort.

Clinical Information	n (%)
Cases	32 (100)
Cores	95 (100)
Cancer	62 (65.2)
Normal tissue (control)	16 (16.8)
Metastases	17 (17.9)
Median follow-up time (months)	100.5
Age (years)	
Mean	59.52
Range	37–79
Disease course	
Alive	46 (48.4)
Dead	24 (25.3)
Unknown	25 (26.3)
Cases with distant metastases	
Yes	9 (28.1)
No	22 (67.8)
Cases with lymph node metastases	
N2	1 (3.1)
N1	2 (6.25)
N0/Nx	28 (88)
Pathology diagnosis	
Clear cell-type RCC	46 (74.2)
Clear- and pseudosarcomatous-type RCC	2 (3.2)
Collecting duct-type RCC	2 (3.2)
Clear cell- and granular cell-type RCC	5 (8.1)
Granular cell-type	4 (6.5)
Sarcomatoid renal cell carcinoma	2 (3.2)
Capsule invasion	
Yes	37 (59.7)
No	22 (35.5)
Unknown	3 (4.8)
Vein invasion	
Yes	16 (25.8)
No	43 (69.4)
Unknown	3 (4.8)
Tumor size (cm)	
Mean	7.5
Range	2.5–17

**Table 2:** Relationship between TOLLIP expression and RCC clinicopathological characteristics.

Variables	Number (%)	TOLLIP		*p*-Value
		High	Low	
		n = 661	n = 216	
Gender				
Females	285	219 (76.84%)	66 (23.16%)	0.0005
Males	590	440 (74.58%)	150 (25.42%)	
Age				
≤60	439	344 (78.36%)	95 (21.64%)	0.206
>60	435	314 (72.18%)	121 (27.82%)	
Stage				
I	451	367 (81.37%)	84 (18.63%)	0.0001
II	102	79 (77.45%)	23 (22.55%)	
III	187	129 (68.98%)	58 (31.02%)	
IV	103	55 (53.4%)	48 (46.6%)	

**Table 3:** Correlations between the expression of autophagy-associated genes and TOLLIP [16,17,18,19,20,21,22,23,24,25,26,27,28,29,30,31,32].

Gene	SQSTM1	NBR1	MAP1LC3	BECN1	TMEM59	ATG13	IRAK1	STAT3	PIK3C3
Correlation coefficient	0.12 *	−0.04	0.18 *	−0.07 *	−0.09 *	0.33 *	−0.07 *	−0.32 *	−0.05
Gene	TAX1BP1	KEAP1	RB1CC1	TRPM3	CELA3B	MTOR	AKT1	ATG9A	VMP1
Correlation coefficient	0.27 *	0.22 *	−0.04	0.14 *	−0.056	0.14 *	0.41 *	−0.09 *	−0.29 *

* *p* < 0.05.

## Data Availability

The data presented in this study are available on request from the corresponding author. The data are not publicly available due to ethical restrictions. The datasets from the TCGA database are publicly available; references [16,17,18,19,20,21,22,23,24,25,26,27,28,29,30,31,32,33].

## References

[1] Siegel R.L., Miller K.D., Jemal A. (2019). Cancer Statistics, 2019. CA Cancer J. Clin..

[2] Jonasch E., Walker C.L., Rathmell W.K. (2021). Clear Cell Renal Cell Carcinoma Ontogeny and Mechanisms of Lethality. Nat. Rev. Nephrol..

[3] Padala S.A., Barsouk A., Thandra K.C., Saginala K., Mohammed A., Vakiti A., Rawla P., Barsouk A. (2020). Epidemiology of Renal Cell Carcinoma. World J. Oncol..

[4] Jones T.M., Carew J.S., Nawrocki S.T. (2020). Therapeutic Targeting of Autophagy for Renal Cell Carcinoma Therapy. Cancers.

[5] D’Avella C., Abbosh P., Pal S.K., Geynisman D.M. (2020). Mutations in Renal Cell Carcinoma. Urol. Oncol..

[6] TOLLIP Toll Interacting Protein [Homo Sapiens (Human)]—Gene—NCBI. https://www.ncbi.nlm.nih.gov/gene?Db=gene&Cmd=ShowDetailView&TermToSearch=54472.

[7] Capelluto D.G.S. (2012). Tollip: A Multitasking Protein in Innate Immunity and Protein Trafficking. Microbes Infect..

[8] Li X., Goobie G.C., Zhang Y. (2021). Toll-Interacting Protein Impacts on Inflammation, Autophagy, and Vacuole Trafficking in Human Disease. J. Mol. Med..

[9] Burns K., Clatworthy J., Martin L., Martinon F., Plumpton C., Maschera B., Lewis A., Ray K., Tschopp J., Volpe F. (2000). Tollip, a New Component of the IL-1RI Pathway, Links IRAK to the IL-1 Receptor. Nat. Cell Biol..

[10] Ito Y., Schaefer N., Sanchez A., Francisco D., Alam R., Martin R.J., Ledford J.G., Stevenson C., Jiang D., Li L. (2018). Toll-Interacting Protein, Tollip, Inhibits IL-13-Mediated Pulmonary Eosinophilic Inflammation in Mice. J. Innate Immun..

[11] Saha S.S., Singh D., Raymond E.L., Ganesan R., Caviness G., Grimaldi C., Woska J.R., Mennerich D., Brown S.-E., Mbow M.L. (2015). Signal Transduction and Intracellular Trafficking by the Interleukin 36 Receptor. J. Biol. Chem..

[12] Zhang G., Ghosh S. (2002). Negative Regulation of Toll-like Receptor-Mediated Signaling by Tollip. J. Biol. Chem..

[13] Zhu L., Wang L., Luo X., Zhang Y., Ding Q., Jiang X., Wang X., Pan Y., Chen Y. (2012). Tollip, an Intracellular Trafficking Protein, Is a Novel Modulator of the Transforming Growth Factor-β Signaling Pathway. J. Biol. Chem..

[14] Bulut Y., Faure E., Thomas L., Equils O., Arditi M. (2001). Cooperation of Toll-like Receptor 2 and 6 for Cellular Activation by Soluble Tuberculosis Factor and Borrelia Burgdorferi Outer Surface Protein A Lipoprotein: Role of Toll-Interacting Protein and IL-1 Receptor Signaling Molecules in Toll-like Receptor 2 Signaling. J. Immunol..

[15] Expression of TOLLIP in Renal Cancer—The Human Protein Atlas. https://www.proteinatlas.org/ENSG00000078902-TOLLIP/pathology/renal+cancer.

[16] Expression of SQSTM1 in Renal Cancer—The Human Protein Atlas. https://www.proteinatlas.org/ENSG00000161011-SQSTM1/pathology/renal+cancer.

[17] Expression of NBR1 in Renal Cancer—The Human Protein Atlas. https://www.proteinatlas.org/ENSG00000188554-NBR1/pathology/renal+cancer.

[18] Expression of MAP1LC3B in Renal Cancer—The Human Protein Atlas. https://www.proteinatlas.org/ENSG00000140941-MAP1LC3B/pathology/renal+cancer.

[19] Expression of BECN1 in Renal Cancer—The Human Protein Atlas. https://www.proteinatlas.org/ENSG00000126581-BECN1/pathology/renal+cancer.

[20] Expression of TMEM59 in Renal Cancer—The Human Protein Atlas. https://www.proteinatlas.org/ENSG00000116209-TMEM59/pathology/renal+cancer.

[21] Expression of IRAK1 in Renal Cancer—The Human Protein Atlas. https://www.proteinatlas.org/ENSG00000184216-IRAK1/pathology/renal+cancer.

[22] Expression of STAT3 in Renal Cancer—The Human Protein Atlas. https://www.proteinatlas.org/ENSG00000168610-STAT3/pathology/renal+cancer.

[23] Expression of PIK3C3 in Renal Cancer—The Human Protein Atlas. https://www.proteinatlas.org/ENSG00000078142-PIK3C3/pathology/renal+cancer.

[24] Expression of TAX1BP1 in Renal Cancer—The Human Protein Atlas. https://www.proteinatlas.org/ENSG00000106052-TAX1BP1/pathology/renal+cancer.

[25] Expression of KEAP1 in Renal Cancer—The Human Protein Atlas. https://www.proteinatlas.org/ENSG00000079999-KEAP1/pathology/renal+cancer.

[26] Expression of RB1CC1 in Renal Cancer—The Human Protein Atlas. https://www.proteinatlas.org/ENSG00000023287-RB1CC1/pathology/renal+cancer.

[27] Expression of TRPM3 in Renal Cancer—The Human Protein Atlas. https://www.proteinatlas.org/ENSG00000083067-TRPM3/pathology/renal+cancer.

[28] Expression of CELA3B in Renal Cancer—The Human Protein Atlas. https://www.proteinatlas.org/ENSG00000219073-CELA3B/pathology/renal+cancer.

[29] Expression of MTOR in Renal Cancer—The Human Protein Atlas. https://www.proteinatlas.org/ENSG00000198793-MTOR/pathology/renal+cancer.

[30] Expression of AKT1 in Renal Cancer—The Human Protein Atlas. https://www.proteinatlas.org/ENSG00000142208-AKT1/pathology/renal+cancer.

[31] Expression of ATG9A in Renal Cancer—The Human Protein Atlas. https://www.proteinatlas.org/ENSG00000198925-ATG9A/pathology/renal+cancer.

[32] Expression of VMP1 in Renal Cancer—The Human Protein Atlas. https://www.proteinatlas.org/ENSG00000062716-VMP1/pathology/renal+cancer.

[33] Begka C., Pattaroni C., Mooser C., Nancey S., McCoy K.D., Velin D., Maillard M.H., Swiss IBD Cohort Study Group (2020). Toll-Interacting Protein Regulates Immune Cell Infiltration and Promotes Colitis-Associated Cancer. iScience.

[34] Chen Y., Choong L.-Y., Lin Q., Philp R., Wong C.-H., Ang B.-K., Tan Y.-L., Loh M.-C.-S., Hew C.-L., Shah N. (2007). Differential Expression of Novel Tyrosine Kinase Substrates during Breast Cancer Development. Mol. Cell. Proteomics.

[35] Zhang T.-M., Liao L., Yang S.-Y., Huang M.-Y., Zhang Y.-L., Deng L., Hu S.-Y., Yang F., Zhang F.-L., Shao Z.-M. (2022). TOLLIP-Mediated Autophagic Degradation Pathway Links the VCP-TMEM63A-DERL1 Signaling Axis to Triple-Negative Breast Cancer Progression. Autophagy.

[36] Zhang Y., Lee C., Geng S., Li L. (2019). Enhanced Tumor Immune Surveillance through Neutrophil Reprogramming due to Tollip Deficiency. JCI Insight.

[37] Schaunaman N., Dimasuay K.G., Kraft M., Chu H.W. (2022). Tollip Interaction with STAT3: A Novel Mechanism to Regulate Human Airway Epithelial Responses to Type 2 Cytokines. Respir. Res..

[38] Wu L., Shen B., Li J., Zhang H., Zhang K., Yang Y., Zu Z., Shen D., Luo M. (2022). STAT3 Exerts pro-Tumor and Anti-Autophagy Roles in Cervical Cancer. Diagn. Pathol..

[39] Huang L., Yang Q., Chen H., Wang Z., Liu Q., Ai S. (2022). Tollip Promotes Hepatocellular Carcinoma Progression via PI3K/AKT Pathway. Open Med..

[40] Alvarez-Meythaler J.G., Garcia-Mayea Y., Mir C., Kondoh H., LLeonart M.E. (2020). Autophagy Takes Center Stage as a Possible Cancer Hallmark. Front. Oncol..

[41] Liberti M.V., Locasale J.W. (2016). The Warburg Effect: How Does It Benefit Cancer Cells?. Trends Biochem. Sci..

[42] Kim T.W., Lee S.Y., Kim M., Cheon C., Ko S.-G. (2018). Kaempferol Induces Autophagic Cell Death via IRE1-JNK-CHOP Pathway and Inhibition of G9a in Gastric Cancer Cells. Cell Death Dis..

[43] Lu K., Psakhye I., Jentsch S. (2014). Autophagic Clearance of polyQ Proteins Mediated by Ubiquitin-Atg8 Adaptors of the Conserved CUET Protein Family. Cell.

[44] Lin C.-Y., Nozawa T., Minowa-Nozawa A., Toh H., Hikichi M., Iibushi J., Nakagawa I. (2020). Autophagy Receptor Tollip Facilitates Bacterial Autophagy by Recruiting Galectin-7 in Response to Group A Streptococcus Infection. Front. Cell. Infect. Microbiol..

[45] Shah J.A., Emery R., Lee B., Venkatasubramanian S., Simmons J.D., Brown M., Hung C.F., Prins J.M., Verbon A., Hawn T.R. (2019). TOLLIP Deficiency Is Associated with Increased Resistance to Legionella Pneumophila Pneumonia. Mucosal Immunol..

[46] Ryan T.A., Phillips E.O., Collier C.L., Jb Robinson A., Routledge D., Wood R.E., Assar E.A., Tumbarello D.A. (2020). Tollip Coordinates Parkin-Dependent Trafficking of Mitochondrial-Derived Vesicles. EMBO J..

[47] Baker B., Geng S., Chen K., Diao N., Yuan R., Xu X., Dougherty S., Stephenson C., Xiong H., Chu H.W. (2015). Alteration of Lysosome Fusion and Low-Grade Inflammation Mediated by Super-Low-Dose Endotoxin. J. Biol. Chem..

[48] Zheng Q., Liu Z., Shen H., Hu X., Zhao M. (2021). Protective Effect of Toll-Interacting Protein Overexpression against Paraquat-Induced Lung Injury in Mice and A549 Cells through Inhibiting Oxidative Stress, Inflammation, and NF-κB Signaling Pathway. Respir. Physiol. Neurobiol..

[49] Kowalski E.J.A., Li L. (2017). Toll-Interacting Protein in Resolving and Non-Resolving Inflammation. Front. Immunol..

[50] Spratt D.E. (2018). Are We Inadvertently Widening the Disparity Gap in Pursuit of Precision Oncology?. Br. J. Cancer.

[51] Lo Y.-L.S., Beckhouse A.G., Boulus S.L., Wells C.A. (2009). Diversification of TOLLIP Isoforms in Mouse and Man. Mamm. Genome.

[52] Chavez-Dominguez R., Perez-Medina M., Lopez-Gonzalez J.S., Galicia-Velasco M., Aguilar-Cazares D. (2020). The Double-Edge Sword of Autophagy in Cancer: From Tumor Suppression to Pro-Tumor Activity. Front. Oncol..

[53] Wu P., Liu J.-L., Pei S.-M., Wu C.-P., Yang K., Wang S.-P., Wu S. (2018). Integrated Genomic Analysis Identifies Clinically Relevant Subtypes of Renal Clear Cell Carcinoma. BMC Cancer.

[54] Yang W., Ju J.-H., Lee K.-M., Nam K., Oh S., Shin I. (2013). Protein Kinase B/Akt1 Inhibits Autophagy by down-Regulating UVRAG Expression. Exp. Cell Res..

[55] Wang P., Kou D., Le W. (2020). Roles of VMP1 in Autophagy and ER-Membrane Contact: Potential Implications in Neurodegenerative Disorders. Front. Mol. Neurosci..

[56] Wesselborg S., Stork B. (2015). Autophagy Signal Transduction by ATG Proteins: From Hierarchies to Networks. Cell. Mol. Life Sci..

[57] Chang Y.-Y., Neufeld T.P. (2009). An Atg1/Atg13 Complex with Multiple Roles in TOR-Mediated Autophagy Regulation. Mol. Biol. Cell.

[58] Zellner S., Behrends C. (2021). Autophagosome Content Profiling Reveals Receptor-Specific Cargo Candidates. Autophagy.

[59] You L., Wang Z., Li H., Shou J., Jing Z., Xie J., Sui X., Pan H., Han W. (2015). The Role of STAT3 in Autophagy. Autophagy.

[60] Yu M., Zeng M., Pan Z., Wu F., Guo L., He G. (2020). Discovery of Novel akt1 Inhibitor Induces Autophagy Associated Death in Hepatocellular Carcinoma Cells. Eur. J. Med. Chem..

[61] Vaccaro M.I., Ropolo A., Grasso D., Iovanna J.L. (2008). A Novel Mammalian Trans-Membrane Protein Reveals an Alternative Initiation Pathway for Autophagy. Autophagy.

[62] Lin W., Sun Y., Qiu X., Huang Q., Kong L., Lu J.J. (2021). VMP1, a Novel Prognostic Biomarker, Contributes to Glioma Development by Regulating Autophagy. J. Neuroinflammation.

[63] Marinković M., Šprung M., Buljubašić M., Novak I. (2018). Autophagy Modulation in Cancer: Current Knowledge on Action and Therapy. Oxid. Med. Cell. Longev..

[64] Heidegger I., Pircher A., Pichler R. (2019). Targeting the Tumor Microenvironment in Renal Cell Cancer Biology and Therapy. Front. Oncol..

[65] Janku F., McConkey D.J., Hong D.S., Kurzrock R. (2011). Autophagy as a Target for Anticancer Therapy. Nat. Rev. Clin. Oncol..

[66] Bouhamdani N., Comeau D., Cormier K., Turcotte S. (2019). STF-62247 Accumulates in Lysosomes and Blocks Late Stages of Autophagy to Selectively Target von Hippel-Lindau-Inactivated Cells. Am. J. Physiol. Cell Physiol..

[67] Li F., Ma Z., Guan Z., Chen Y., Wu K., Guo P., Wang X., He D., Zeng J. (2015). Autophagy Induction by Silibinin Positively Contributes to Its Anti-Metastatic Capacity via AMPK/mTOR Pathway in Renal Cell Carcinoma. Int. J. Mol. Sci..

[68] Sun H., Wang W., Che Y., Jiang X. (2016). Fungal Secondary Metabolites Rasfonin Induces Autophagy, Apoptosis and Necroptosis in Renal Cancer Cell Line. Mycology.

[69] He Y.-H., Tian G. (2020). Autophagy as a Vital Therapy Target for Renal Cell Carcinoma. Front. Pharmacol..

[70] White E. (2012). Deconvoluting the Context-Dependent Role for Autophagy in Cancer. Nat. Rev. Cancer.

[71] Bray K., Mathew R., Lau A., Kamphorst J.J., Fan J., Chen J., Chen H.-Y., Ghavami A., Stein M., DiPaola R.S. (2012). Autophagy Suppresses RIP Kinase-Dependent Necrosis Enabling Survival to mTOR Inhibition. PLoS ONE.

[72] Escudier B., Porta C., Schmidinger M., Rioux-Leclercq N., Bex A., Khoo V., Grünwald V., Gillessen S., Horwich A., ESMO Guidelines Committee (2019). Renal Cell Carcinoma: ESMO Clinical Practice Guidelines for Diagnosis, Treatment and Follow-Up. Ann. Oncol..

[73] Nathan P., Chao D., Brock C., Savage P., Harries M., Gore M., Eisen T. (2006). The Place of VEGF Inhibition in the Current Management of Renal Cell Carcinoma. Br. J. Cancer.

[74] Zheng B., Zhu H., Gu D., Pan X., Qian L., Xue B., Yang D., Zhou J., Shan Y. (2015). MiRNA-30a-Mediated Autophagy Inhibition Sensitizes Renal Cell Carcinoma Cells to Sorafenib. Biochem. Biophys. Res. Commun..

[75] Pal K., Madamsetty V.S., Dutta S.K., Wang E., Angom R.S., Mukhopadhyay D. (2019). Synchronous Inhibition of mTOR and VEGF/NRP1 Axis Impedes Tumor Growth and Metastasis in Renal Cancer. NPJ Precis. Oncol..

[76] Li H., Jin X., Zhang Z., Xing Y., Kong X. (2013). Inhibition of Autophagy Enhances Apoptosis Induced by the PI3K/AKT/mTor Inhibitor NVP-BEZ235 in Renal Cell Carcinoma Cells. Cell Biochem. Funct..

[77] Singla M., Bhattacharyya S. (2017). Autophagy as a Potential Therapeutic Target during Epithelial to Mesenchymal Transition in Renal Cell Carcinoma: An in Vitro Study. Biomed. Pharmacother..

[78] Duran I., Lambea J., Maroto P., González-Larriba J.L., Flores L., Granados-Principal S., Graupera M., Sáez B., Vivancos A., Casanovas O. (2017). Resistance to Targeted Therapies in Renal Cancer: The Importance of Changing the Mechanism of Action. Target. Oncol..

[79] Budczies J., Klauschen F., Sinn B.V., Győrffy B., Schmitt W.D., Darb-Esfahani S., Denkert C. (2012). Cutoff Finder: A Comprehensive and Straightforward Web Application Enabling Rapid Biomarker Cutoff Optimization. PLoS ONE.

